# Disparities in outcomes of COVID-19 hospitalizations in native American individuals

**DOI:** 10.3389/fpubh.2023.1220582

**Published:** 2023-08-15

**Authors:** Christian Bime, Ying Wang, Gordon Carr, Dennis Swearingen, Sherri Kou, Pam Thompson, Vinita Kusupati, Sairam Parthasarathy

**Affiliations:** ^1^Division of Pulmonary, Allergy, Critical Care & Sleep Medicine, Department of Medicine, University of Arizona, Tucson, AZ, United States; ^2^Department of Informatics Technology, Banner Health, Phoenix, AZ, United States; ^3^Department of Medicine, University of Arizona College of Medicine, Tucson, AZ, United States; ^4^Department of Biomedical Informatics, University of Arizona College of Medicine, Phoenix, AZ, United States; ^5^Department of Medical Informatics, Banner Health, Phoenix, AZ, United States; ^6^Department of Academic and Facilities Research, Banner Health, Phoenix, AZ, United States; ^7^Division of General Internal Medicine, Department of Medicine, University of Arizona, Tucson, AZ, United States

**Keywords:** COVID-19, racial disparities, mortality, Native American, ARDS

## Abstract

**Objectives:**

This study aimed to investigate COVID-19-related disparities in clinical presentation and patient outcomes in hospitalized Native American individuals.

**Methods:**

The study was performed within 30 hospitals of the Banner Health system in the Southwest United States and included 8,083 adult patients who tested positive for SARS-CoV-2 infection and were hospitalized between 1 March 2020 and 4 September 2020. Bivariate and multivariate analyses were used to assess racial and ethnic differences in clinical presentation and patient outcomes.

**Results:**

COVID-19-related hospitalizations in Native American individuals were over-represented compared with non-Hispanic white individuals. Native American individuals had fewer symptoms at admission; greater prevalence of chronic lung disease in the older adult; two times greater risk for ICU admission despite being younger; and 20 times more rapid clinical deterioration warranting ICU admission. Compared with non-Hispanic white individuals, Native American individuals had a greater prevalence of sepsis, were more likely to require invasive mechanical ventilation, had a longer length of stay, and had higher in-hospital mortality.

**Conclusion:**

Native American individuals manifested greater case-fatality rates following hospitalization than other races/ethnicities. Atypical symptom presentation of COVID-19 included a greater prevalence of chronic lung disease and a more rapid clinical deterioration, which may be responsible for the observed higher hospital mortality, thereby underscoring the role of pulmonologists in addressing such disparities.

## Introduction

The coronavirus 2019 (COVID-19) pandemic has been a major threat to public health. As of August 2022, the number of confirmed SARS-CoV-2 infections in the United States has reached 93.7 million with a death toll surpassing 1 million individuals (1). COVID-19 has unevenly impacted different populations (2, 3). Vulnerable groups such as the older adults, disabled, and racial minorities have been more severely affected (3, 4). Studies have shown that African Americans were disproportionally affected by COVID-19 with greater rates of hospitalization and death than non-Hispanic white individuals (3, 5). Such health disparities in the African American community were associated with social determinants of health, including lower income, lack of health insurance, poor access to care, crowded housing, lack of independent transportation, and greater exposure to COVID-19 (6). Native Americans and Hispanic individuals suffer a greater burden of chronic medical illnesses, such as diabetes, obesity, and heart disease, which, in turn, may confer a greater risk for COVID-19 (7, 8). However, few studies have examined the COVID-19 hospitalization and mortality rates in Native American populations (9–11). A recent systematic review and meta-analysis stated that there was insufficient evidence in Native Americans to draw strong conclusions regarding disparities in COVID-19 and that more research was needed (12). Moreover, they concluded that the increased susceptibility to COVID-19 alone does not seem to explain the observed disparities in other minority populations (African American individuals and Hispanic individuals) (12). Perhaps barriers to healthcare access and delayed medical attention may be responsible for the observed disparities. The unique challenges faced by Native American populations are well documented and could explain the increased risk of COVID-19 morbidity and mortality (13–15). In addition to having a higher burden of risk factors for severe COVID-19 disease (15), poor access to tertiary care centers could lead to a delay in presentation, hence poorer outcomes (13). We explore the preventable differences in hospitalization including the burden of intensive care unit (ICU) admissions across such race/ethnicities and explore excess hospital mortality, which are defined as constituting health disparities by the National Institutes of Health for Minority Health and Disparities (NIMHD) and Centers for Disease Control (CDC) (16, 17).

Moreover, although African American and Hispanic populations experience disproportionately greater rates of severe acute respiratory syndrome coronavirus-2 (SARS-CoV-2) infection and COVID-19-related mortality, case-fatality rates were similar in non-Hispanic white individuals (12). But inadequate adjustment for the acuity of illness or inter-hospital transfers—which are major determinants of in-hospital mortality—may have prevented the detection of differences in case-fatality rates as the studies in Native Americans were derived primarily from hospital reporting of deaths to the CDCl (9).

The objective of this study was to investigate COVID-19-related disparities in clinical presentation and patient outcomes in the Native American population. Additionally, the study aimed to assess the case-fatality rates after fully adjusting for the acuity of illness and inter-hospital transfers in minority populations with COVID-19 requiring hospitalization.

## Methods

### Study design, setting, and population

This was a retrospective observational cohort study conducted within the Banner Health system (30 acute care hospitals) in the Southwest United States. Banner Health is a large nationally recognized health system operating in six states in the United States: Arizona, California, Nebraska, Nevada, Wyoming, and Colorado. Most of the hospitals are located in Arizona, including three academic medical centers: Banner—University Medical Center Tucson, Banner—University Medical Center Phoenix, and Banner—University Medical Center South. Banner Health provides healthcare for a large segment of the Native American population in Arizona and neighboring states in the mountain west of the United States.

The study included adult (≥18) patients who tested positive for infection with SARS-CoV-2 using a qualitative polymerase-chain-reaction assay and were hospitalized between 1 March 2020 and 4 September 2020. We obtained data prior to the availability of the COVID-19 vaccine in order to avoid the effects of vaccine hesitancy, vaccination rates, monoclonal antibodies, and routine administration of steroids and remdesivir on observed disparities (18–20). The Institutional Review Board of Banner Health (IRB#483-20-0076) approved this study.

### Data collection

Data were retrospectively collected from the electronic medical records (EMR) stored in a data warehouse (Banner Health Enterprise Data Warehouse). Data included demographics (age, sex, and race), health insurance, patient self-reported address, admission source, ICU utilization, timestamps of registration and ICU admission, timestamps of discharge from ICU and hospital, discharge disposition (alive or expired), and use of invasive mechanical ventilation. Self-reported ethnicity in the EMR system was used to identify the Hispanic population, and self-reported race information was used to identify other racial groups. Racial groups in this study include non-Hispanic white, Hispanic, Black or African American, Native American, Asian, Middle Eastern Indian, Native Hawaiian, or Other Pacific Islander. To ensure sufficient sample size in each comparator group, we collapsed cases into the following five race/ethnicity groups: non-Hispanic white, Hispanic, Black, Native American, and others. The median income for zip codes of patient residence was used as a surrogate for socioeconomic status. Insurance plans were categorized into five groups: Private, Medicaid, Medicare, Self-Pay, and others (Supplementary Table 1). Chronic conditions were collected based on previous ICD10 diagnosis codes related to any type of encounter in the last 5 years within the Banner delivery network (Supplementary Table 2). COVID-19 symptoms such as cough, fever, dyspnea, abdominal symptoms, and myalgia were extracted from an unstructured text field “chief complaint” from the patient's records at the beginning of visits.

### Statistical analysis

We compared in-hospital outcomes of patients with COVID-19 by race/ethnicity groups. Outcome (dependent) variables included in-hospital mortality, invasive mechanical ventilation, ICU admission, and inter-hospital transfer vs. admission through ED, ICU LOS, and hospital LOS. Time (hours) from entering the hospital (“registration”) to ICU admission and ICU LOS were also deemed outcome variables in a subgroup of patients requiring ICU admission. Time from registration to ICU admission was examined to reflect how quickly a patient's condition deteriorated. Both hospital LOS and ICU LOS were investigated in live discharges. Covariates included various chronic medical conditions, insurance status, type of insurance, median income imputed from ZIP code of residence, age, sex, admission through ED or inter-hospital transfer, and symptom presentation (cough, fever, dyspnea, and abdominal symptoms). Bivariate analyses were used to characterize patients across the racial groups. Continuous variables were expressed using median and interquartile range (IQR). Categorical variables were described with frequencies and percentages. Univariate logistic regression was used to examine distributions of categorical variables among race/ethnicity groups, and ANOVA was used for examining continuous variables among race/ethnicity groups. The chi-square tests and Student's *t*-tests were used for *post-hoc* analyses. Two-sided exact *P*-values were reported, and *P*-values of ≤ 0.05 were considered statistically significant. *P*-values were adjusted by multiplicity with Bonferroni adjustment. Four strata by age group (18–39, 40–59, 60–79, and ≥80 years) were created to age-stratify distributions of chronic conditions, insurance plans, and outcome factors.

Multivariable logistic regression and linear regression were performed to examine factors associated with outcome variables in both hospitalized patients and ICU patients. Collinearity was determined among covariates using the Pearson correlation and variance inflation factor (VIF) methods (Supplementary Tables 3, 4 for inpatients, Supplementary Tables 5, 6 for ICU patients). The criteria (21) for selecting covariates of the model were as follows: (1) All covariates with *P* of < 0.25 in the bivariate analyses were excluded (22). (2) Pearson correlation coefficients <0.75 and VIF <5 for each covariate were included (23). The variable for the total number of chronic conditions was removed from the regression analysis based on the guidance for controlling confounders suggested by Lederer et al. (21). Regression assumptions such as linearity, homoscedasticity, and normality of error terms were checked before performing univariate and multivariate regressions. Median income values were missing in 12.3% of individuals due to a lack of residential address. An imputation with a sample median was used to replace the missing values. All analyses were performed using Python 3.6.2 (Python Software Foundation, Beaverton, OR).

## Results

In total, there were 8,083 inpatients with COVID-19 (≥18 years), 7,515 (92.9%) were in Arizona. In such COVID-19 inpatients, when compared to the proportion of race/ethnicity populations in Arizona (2019 census) (24), the minority race/ethnicities—Native American (7.5%), Black (6.0%), and Hispanic individuals (40.6%)—were over-represented (all *P* < 0.0001), while non-Hispanic white individuals (41.7%) were under-represented (*P* < 0.0001, Supplementary Figure 1).

### Characteristics of hospitalized patients by race using bivariate analysis

#### Age, sex, and socioeconomic status

Among 8,083 hospitalized patients with COVID-19, the median age of patients was 60 years (interquartile range [IQR] 46–73) and 50.4% were male patients. Native American, Black, and Hispanic patients were younger than the non-Hispanic white patients (Table 1). Of patients <60 years, 67.6% were Native American, 58.8% Hispanic, and 55.6% Black, compared with 34.8% of non-Hispanic white individuals (Supplementary Figure 2A). The median income of Native Americans was lower than non-Hispanic white individuals (Table 1; *P* < 0.0001). Native American patients were 3.5-fold more likely to be on Medicaid insurance than non-Hispanic white individuals (51.75 vs. 14.3%, Table 1; *P* < 0.0001). In age-stratified data, Native American patients aged 18–39 years had the highest proportion of Medicaid coverage among all age categories (Supplementary Figure 2B, *P* < 0.001). Hispanic (33.6%) and Black patients (28.5%) had higher rates of Medicaid coverage than non-Hispanic white individuals (14.2%) (Table 1; *P* < 0.0001).

**Table 1:** Characteristics of hospitalized COVID-19 patients, overall and by race.

**Characteristics**	**Overall sample (*n =* 8,083)**	**Native American (*n =* 570, 7.05%)**	**Black (*n =* 471, 5.83%)**	**Hispanic (*n =* 3,305, 40.89%)**	**Others (*n =* 344, 4.26%)**	**Non-Hispanic white (*n =* 3,393, 41.98%)**	***P*-values^$^**
Age (years), Median (IQR)	60 (46–73)	50 (38–63)^†^	57 (42–67)^@^	55 (43–68)^‡^	61 (49–72)	67 (54–78)	
18–39 years, *n* (%)	1,357 (16.8%)	163 (28.6%)	103 (21.9%)	663 (20.1%)	45 (13.1%)	383 (11.3%)	<0.0001
40–59 years, *n* (%)	2,565 (31.7%)	222 (39.0%)	159 (33.8%)	1,279 (38.7%)	109 (31.7%)	796 (23.5%)	
60–79 years, *n* (%)	3,047 (37.7%)	158 (27.7%)	176 (37.4%)	1,116 (33.8%)	147 (42.7%)	1,450 (42.7%)	
>=80 years, *n* (%)	1,114 (13.8%)	27 (4.7%)	33 (7.0%)	247 (7.5%)	43 (12.5%)	764 (22.5%)	
Male, *n* (%)	4,075 (50.4%)	255 (44.7%)	233 (49.5%)	1,646 (49.8%)	170 (49.4%)	1,771 (52.2%)	0.01
Median income ($), Median (IQR)	$48,559 (38,373–53,981)	$40,083 (26,542–48,559)^†^	$48,559 (39,504–55,156)	$48,559 (37,391–51,915)	$48,559 (37,696- 52,234)	$48,999 (43,738–60,756)	<0.0001
Insurance, *n* (%)							
Medicaid	2,107 (26.1%)	295 (51.8%)^†^	134 (28.5%)^@^	1,111 (33.6%)^‡^	84 (24.4%)	483 (14.2%)	<0.0001
Medicare	3,443 (42.6%)	155 (27.2%)	189 (40.1%)	1,040 (31.5%)	135 (39.2%)	1,924 (56.7%)	
Others	18 (0.2%)	1 (0.2%)	1 (0.2%)	5 (0.2%)	0 (0.0%)	11 (0.3%)	
Private	1,902 (23.5%)	95 (16.7%)	114 (24.2%)	774 (23.4%)	91 (26.5%)	828 (24.4%)	
Self-Pay	613 (7.6%)	24 (4.2%)	33 (7.0%)	375 (11.4%)	34 (9.9%)	147 (4.3%)	
Chronic conditions, n (%)							
Stroke	741 (9.2%)	42 (7.4%)	50 (10.6%)	245 (7.4%)	31 (9.0%)	373 (11.0%)	<0.0001
Chronic kidney	1,757 (21.7%)	112 (19.7%)	151 (32.1%)	549 (16.6%)	63 (18.3%)	882 (26.0%)	<0.0001
Chronic liver	426 (5.3%)	64 (11.2%)	30 (6.4%)	150 (4.5%)	23 (6.7%)	159 (4.7%)	<0.0001
Hypoxemia	5,126 (63.4%)	341 (59.8%)	268 (56.9%)	2,068 (62.6%)	206 (59.9%)	2,243 (66.1%)	<0.0001
Chronic lung	165 (2.0%)	15 (2.6%)	4 (0.9%)	84 (2.5%)	5 (1.5%)	57 (1.7%)	0.02
Coronary heart	1,676 (20.7%)	63 (11.1%)	103 (21.9%)	511 (15.5%)	61 (17.7%)	938 (27.7%)	<0.0001
Diabetes mellitus	3,506 (43.4%)	288 (50.5%)^†^	216 (45.9%)^@^	1,584 (47.9%)^‡^	150 (43.6%)	1,268 (37.4%)	<0.0001
Dementia	718 (8.9%)	17 (3.0%)	37 (7.9%)	162 (4.9%)	25 (7.3%)	477 (14.1%)	<0.0001
Depression	1,755 (21.7%)	77 (13.5%)	99 (21.0%)	528 (16.0%)	48 (14.0%)	1,003 (29.6%)	<0.0001
Dyslipidemia	704 (8.7%)	26 (4.6%)	43 (9.1%)	264 (8.0%)	26 (7.6%)	345 (10.2%)	<0.0001
Hyperlipidemia	3,370 (41.7%)	169 (29.7%)	193 (41.0%)	1,234 (37.3%)	128 (37.2%)	1,646 (48.5%)	<0.0001
Hypertension	966 (12.0%)	49 (8.6%)	51 (10.8%)	273 (8.3%)	36 (10.5%)	557 (16.4%)	<0.0001
Hyperthyroidism	91 (1.1%)	8 (1.4%)	8 (1.7%)	33 (1.0%)	3 (0.9%)	39 (1.2%)	0.65
Hypothyroidism	1,296 (16.0%)	62 (10.9%)	52 (11.0%)	414 (12.5%)	44 (12.8%)	724 (21.3%)	<0.0001
Ischemic heart	512 (6.3%)	19 (3.3%)	36 (7.6%)	156 (4.7%)	21 (6.1%)	280 (8.3%)	<0.0001
Insomnia	523 (6.5%)	27 (4.7%)	31 (6.6%)	158 (4.8%)	19 (5.5%)	288 (8.5%)	<0.0001
Defibrillator	361 (4.5%)	16 (2.8%)	19 (4.0%)	86 (2.6%)	8 (2.3%)	232 (6.8%)	<0.0001
Do not resuscitate status	1,964 (24.3%)	117 (20.5%)	71 (15.1%)	594 (18.0%)	86 (25.0%)	1,096 (32.3%)	<0.0001
Acute myocardial infarction	547 (6.8%)	35 (6.1%)	42 (8.9%)	213 (6.4%)	12 (3.5%)	245 (7.2%)	0.02
Atrial fibrillation	994 (12.3%)	33 (5.8%)	49 (10.4%)	250 (7.6%)	35 (10.2%)	627 (18.5%)	<0.0001
Ventricular fibrillation	341 (4.2%)	21 (3.7%)	32 (6.8%)	93 (2.8%)	13 (3.8%)	182 (5.4%)	<0.0001
Morbid obesity	2,825 (35.0%)	245 (43.0%)^†^	166 (35.2%)	1,187 (35.9%)^‡^	99 (28.8%)	1,128 (33.2%)	<0.0001
Sleep apnea	925 (11.4%)	58 (10.2%)	59 (12.5%)	270 (8.2%)	28 (8.1%)	510 (15.0%)	<0.0001
Obesity hypoventilation	97 (1.2%)	7 (1.2%)	7 (1.5%)	30 (0.9%)	0 (0.0%)	53 (1.6%)	0.03
Total comorbidities, *n* (%)							
0	593 (7.3%)	33 (5.8%)	48 (10.2%)	266 (8.1%)	34 (9.9%)	212 (6.3%)	<0.0001
≤ 3	3,787 (46.9%)	327 (57.4%)	205 (43.5%)	1,795 (54.3%)	178 (51.7%)	1,282 (37.8%)	
>3	3,703 (45.8%)	210 (36.8%)^†^	218 (46.3%)^@^	1,244 (37.6%)^‡^	132 (38.4%)	1,899 (56.0%)	
COVID-19 symptoms, n (%)							
Cough	2,535 (31.4%)	101 (17.7%)^†^	145 (30.8%)	1,225 (37.1%)	81 (23.5%)	983 (29.0%)	<0.0001
Abdominal symptoms	878 (10.9%)	38 (6.7%)^†^	62 (13.2%)	407 (12.3%)	26 (7.6%)	345 (10.2%)	<0.0001
Dyspnea	3,639 (45.0%)	169 (29.7%)^†^	189 (40.1%)	1,684 (51.0%)	135 (39.2%)	1,462 (43.1%)	<0.0001
Fever	2,363 (29.2%)	88 (15.4%)^†^	140 (29.7%)	1,058 (32.0%)	95 (27.6%)	982 (28.9%)	<0.0001
Myalgia	129 (1.6%)	9 (1.6%)	14 (3.0%)	49 (1.5%)	6 (1.7%)	51 (1.5%)	0.19

IQR, interquartile range; *P*-value for ANOVA or univariate logistic regression; *post-hoc* comparisons against non-Hispanic white individuals as comparators (^†^For Native American compared with non-Hispanic white individuals;^‡^For Hispanic individuals again compared with non-Hispanic white individuals; ^@^For Black individuals compared with non-Hispanic white individuals; *post-hoc* comparisons were chi-square test and Student's *t*-test).

#### Comorbidities and clinical symptoms

Diabetes mellitus, obesity, and chronic liver disease were more prevalent in Native Americans than in non-Hispanic white individuals (Table 1; all *P* < 0.0001), and this was consistent across all age categories (all *P* < 0.05, Supplementary Figures 3A–C). The rate of chronic lung disease in Native Americans at age 60–79 years was 2-fold greater than in non-Hispanic white individuals (5.7 vs. 2.3%, *P* = 0.03, Supplementary Figure 3D). Interestingly, the percentage of individuals with multiple chronic medical conditions (>3 chronic medical conditions) was lower in the minority race/ethnicities such as Native American (36.8%), Hispanic (37.6%), and Black individuals (46.3%) than non-Hispanic white individuals (56.0%) (Table 1; *P* < 0.0001). The predominant presenting symptom was dyspnea (45%), and in descending order were cough (31%), fever (29%), abdominal complaints (11%), and myalgia (1.6%; Table 1). Excepting for fever, Native Americans had lower rates of other symptomatology than non-Hispanic white individuals (all *P* < 0.05; Table 1).

### Characteristics of COVID-19 outcomes by race and age using bivariate analysis

#### Sepsis, ICU admission, mechanical ventilation, and hospital mortality

**Figure 1:**
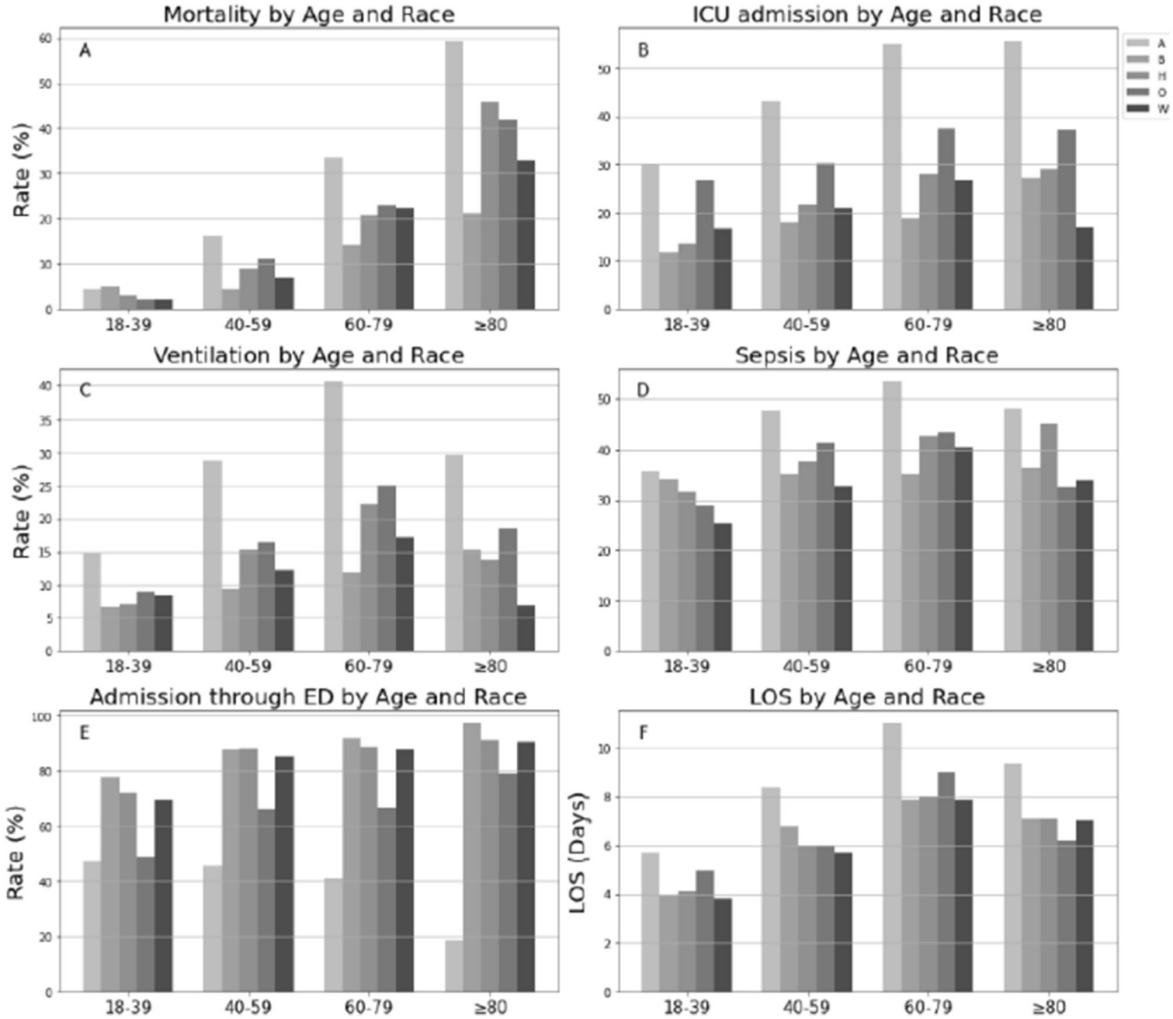
Distributions of clinical outcomes of COVID-19 by age and race in hospitalized population. LOS was examined in live inpatients; A, Native American; B, Black and African American; H, Hispanic; O, Others; W, White. **(A)** Mortality by Age and Race. **(B)** ICU admission by Age and Race. **(C)** Ventilation by Age and Race. **(D)** Sepis by Age and Race. **(E)** Admission through ED by Age and Race. **(F)** LOS by Age and Race.

Overall, in-hospital mortality rate for COVID-19 patients was 16.5% (Table 2) and progressively increased across age (from 3% in 18–39 years of age to 36.4% in individuals ≥80 years; 18–29 vs. 40–59, chi-square = 88.9; 40–59 vs. 60–79, chi-square=88.9, 60–79 vs. ≥80, chi-square=88.9; all Bonferroni-adjusted *P* < 0.0001, Figure 1A). Native Americans had the highest rate of ICU admissions (43.3%) among all race/ethnicity groups (Native vs. Black, chi-square = 23.9; Native vs. Hispanic, chi-square = 23.9; Native vs. white, chi-square=23.9; Native vs. Others, chi-square=23.9; all Bonferroni-adjusted *P* < 0.0001, Table 2). This pattern was consistent across all age categories (all *P* < 0.0001, Figure 1B). Approximately 50% of Native American patients >60 years were admitted to the ICU. Greater than 15% of patients needed invasive mechanical ventilation, and both Native Americans (28.1%) and Hispanic individuals (15.9%) had higher rates of invasive mechanical ventilation than non-Hispanic white individuals (12.7%). Native Americans belonging to the age category (60–79 years) had the highest rate of mechanical ventilation compared with any other race/ethnicity age group categories (*P* < 0.05, Figure 1C; Supplemetnary Table 7). Native Americans had higher rates of sepsis than Black individuals (chi-square = 2.11, Bonferroni-adjusted *P* < 0.001), Hispanic individuals, or non-Hispanic white individuals (chi-square = 2.11, Bonferroni-adjusted *P* < 0.0001) (Table 2; Figure 1D). Approximately 50% of Native American patients >40 years developed sepsis (Figure 1D).

**Table 2:** Characteristics of COVID-19 patients' outcomes by race.

**Characteristics**	**Overall sample (*N =* 8,083)**	**Native American (*n =* 570, 7.05%)**	**Black (*n =* 471, 5.83%)**	**Hispanic (*n =* 3,305, 40.89%)**	**Others (*n =* 344, 4.26%)**	**Non-Hispanic white (*n =* 3393, 41.98%)**	***P*-values^$^**
Clinical outcomes, *n* (%)							
Type of admission through the emergency, *n* (%)	6,604 (81.7%)	248 (43.5%)	413 (87.7%)	2,815 (85.2%)	226 (65.7%)	2,902 (85.5%)	<0.0001
Admission by inter-hospital transfer, *n* (%)	446 (5.52%)	168 (29.5%)^†^	11 (2.3%)	116 (3.5%)	44 (12.8%)	107 (3.2%)	<0.0001
In-hospital mortality, *n* (%)	1,333 (16.5%)	112 (19.7%)^†^	44 (9.3%)	476 (14.4%)	65 (18.9%)	636 (18.7%)	<0.0001
Use of invasive mechanical ventilation, *n* (%)	1,231 (15.2%)	160 (28.1%)^†^	48 (10.2%)	525 (15.9%)^‡^	67 (19.5%)	431 (12.7%)	<0.0001
Acute complication as sepsis, *n* (%)	3,045 (37.7%)	261 (45.8%)^†^	165 (35.0%)	1,281 (38.8%)	136 (39.5%)	1,202 (35.4%)	<0.0001
ICU admission, *n* (%)	1,942 (24.0%)	247 (43.3%)^†^	83 (17.6%)	752 (22.8%)	116 (33.7%)	744 (21.9%)	<0.0001
Total hospital length of stay (days), median (IRQ)	6.35 (3.7–11.9)	13.69 (7.1–24.0)^†^	11.84 (7.2–15.8)^@^	13.15 (6.1–20.8)^‡^	16.0 (5.4–25.6)	8.9 (4.8–14.6)	<0.0001

IQR, interquartile range; *P*-value for ANOVA or univariate logistic regression; *post-hoc* comparisons against non-Hispanic white individuals as comparators (^†^For Native American compared with non-Hispanic white individuals;^‡^For Hispanic individuals again compared with non-Hispanic white individuals; ^@^ For Black individuals compared with non-Hispanic white individuals; *post-hoc* comparisons were chi-square test and Student's *t*-test).

#### Admission through ED and hospital stay

Approximately 82% of hospitalized patients were admitted through the ED (85.2% Hispanic individuals, 87.7% Black individuals, 85.5% white individuals, and 43.5 Native American individuals) (Table 1). Native American individuals were less likely to be admitted through the ED or conversely more likely to undergo inter-hospital transfers (*P*-values <0.0001, Figure 1E). The median hospital LOS was 6.4 days for all inpatients (Table 1). LOS in Native American individuals was significantly longer than in non-Hispanic white individuals (Table 1). Native American individuals who were <80 years old had a longer LOS than non-Hispanic white individuals (all *P* < 0.0001; Table 3), and Native American patients who were between 40 and 69 years of age had the longest LOS of any age group (all *P* < 0.05, Figure 1F).

**Table 3:** Multivariable logistic and linear regression results for factors associated with hospital death, mechanical intervention, ICU admission, acute complication as sepsis, LOS, admitted through ED in hospitalized patients.

	**Hospital mortality**		**Invasive mechanical ventilation**		**ICU admission**		**Acute complication as sepsis**		**Total length of hospital stay**		**Being admitted through ED**	
	**OR (95%CI)**	***P*** **-values**	**OR (95%CI)**	***P*** **-values**	**OR (95%CI)**	***P-*** **values**	**OR (95%CI)**	**P-values**	**Coef (95%CI)**	***P*** **-values**	**OR (95%CI)**	***P*** **-values**
Age (Cont.)	1.0 (1.0–1.0)	<0.0001	1.0 (1.0–1.0)	0.41	1.0 (1.0–1.0)	0.05	1.0 (1.0–1.0)	0.11	0.1 (0.1–0.1)	<0.0001	1.0 (1.0–1.0)	<0.0001
**Race**												
White	RF	NA	RF	NA	RF	NA	RF	NA	RF	NA	RF	NA
Native American	2.3 (1.6–3.2)	<0.0001	2.3 (1.8–3.0)	<0.0001	2.4 (1.9–3.0)	<0.0001	1.7 (1.4–2.8)	<0.0001	2.1 (1.2–3.0)	<0.0001	0.2 (0.1–0.2)	<0.0001
Black	0.9 (0.6–1.4)	0.6	1.0 (0.7–1.3)	0.8	0.9 (0.7–1. 26)	0.4	1.1 (0.9–1.4)	0.4	0.1 (-0.7–1.0)	0.8	1.7 (1.2–2.4)	<0.01
Hispanic	1.4 (1.1–1.7)	<0.01	1.4 (1.2–1.6)	<0.001	1.1 (1.0–1.3)	0.18	1.2 (1.1–1.3)	<0.01	0.5 (0.0–1.0)	<0.05	1.0 (0.9–1.2)	0.8
Others	1.5 (1.0–2.36)	0.03	1.5 (1.1–2.1)	0.01	1.8 (1.4–2.3)	<0.0001	1.3 (1.0–1.6)	0.05	0.5 (-0.6–1.5)	0.4	0.3 (0.2–0.4)	<0.0001
**Insurance**												
Private	RF	NA	RF	NA	RF	NA	RF	NA	RF	NA	RF	NA
Medicaid	1.2 (0.9–1.6)	0.2	1.0 (0.8–1.2)	0.9	1.1 (1.0–1.3)	0.1	1.1 (0.9–1.2)	0.3	0.6 (0.0–1.2)	0.0	1.0 (0.8–1.3)	0.8
Medicare	1.0 (0.8–1.3)	0.9	0.9 (0.7–1.1)	0.3	0.9 (0.8–1.1)	0.3	1.0 (0.8–1.1)	0.6	-0.6 (-1.2–0.1)	0.1	1.2 (1.0–1.5)	0.1
Others	0.4 (0.0–3.9)	0.4	1.3 (0.3–5.4)	0.7	1.1 (0.343–6)	0.9	0.8 (0.3–2.2)	0.6	1.2 (-2.8–5.1)	0.6	1.7 (0.4–6.6)	0.5
Self-Pay	1.2 (0.8–1.9)	0.4	0.9 (0.7–1.3)	0.7	0.9 (0.7–1.2)	0.5	0.9 (0.7–1.1)	0.3	-1.3 (-2.1–0.5)	<0.01	1.4 (1.0–1.9)	<0.05
Male vs. female	1.7 (1.4–2.0)	<0.0001	2.0 (1.7–2.3)	<0.0001	1.7 (1.5–1.9)	<0.0001	1.4 (1.3–1.6)	<0.0001	1.2 (0.8–1.6)	<0.0001	1.3 (1.1–1.5)	<0.01
Standardized income (Cont.)	0.4 (0.2–0.9)	<0.05	0.2 (0.1–0.5)	<0.001	0.3 (0.27–0.6)	<0.001	0.7 (0.4–1.2)	0.2	-3.0 (5.2–0.8)	<0.01	2.0 (0.9–4.4)	0.1
**Chronic conditions**												
Stroke	1.0 (0.8–1.3)	0.7	1.1 (0.9–1.4)	0.5	1.1 (0.9–1.3)	0.5	1.0 (0.8–1.1)	0.6	0.7 (-0.1–1.5)	0.10	1.2 (1.0–1.6)	0.1
Chronic kidney	1.1 (0.9–1.3)	0.5	1.0 (0.8–1.2)	1.0	1.1 (0.9–1.2)	0.4	1.1 (1.0–1.3)	0.1	1.5 (0.9–2.1)	<0.0001	1.17 (1.0–1.4)	0.1
Chronic liver	1.4 (1.0–1.9)	<0.05	0.7 (0.6–1.0)	0.05	0.9 (0.7–1.2)	0.5	0.9 (0.7–1.1)	0.4	-0.4 (-1.3–0.6)	0.5	1.7 (1.2–2.3)	<0.01
Hypoxemia	0.8 (0.7–1.0)	<0.01	0.4 (0.4–0.5)	<0.0001	0.7 (0.7–0.8)	<0.0001	1.3 (1.2–1.4)	<0.0001	0.6 (0.2–1.0)	<0.01	1.0 (0.9–1.2)	0.9
Chronic lung	6.1 (3.9–9.6)	<0.0001	4.2 (2.8–6.1)	<0.0001	3.1 (2.2–4.5)	<0.0001	2.2 (1.5–3.1)	<0.0001	11.9 (9.7–14.0)	<0.0001	1.1 (0.6–1.8)	0.8
Coronary heart	0.9 (0.7–1.17)	0.2	0.6 (0.5–0.8)	<0.0001	0.76 (0.6–0.8)	<0.0001	0.7 (0.6–0.8)	<0.0001	-1.4 (-2.0–0.8)	<0.0001	1.0 (0.8–1.3)	0.9
Diabetes mellitus	1.2 (1.0–1.5)	<0.05	1.4 (1.2–1.6)	<0.0001	1.3 (1.2–1.5)	<0.0001	1.2 (1.1–1.4)	<0.0001	1.5 (1.1–2.0)	<0.0001	1.1 (1.0–1.4)	<0.05
Dementia	0.8 (0.64–1.0)	0.1	0.4 (0.3–0.5)	<0.0001	0.5 (0.4–0.7)	<0.0001	0.9 (0.7–1.0)	0.1	-0.8 (-1.6–0.1)	0.1	1.6 (1.2–2.1)	<0.01
Depression	0.7 (0.6–0.8)	<0.0001	0.7 (0.6–0.9)	<0.001	0.8 (0.7–0.9)	<0.01	0.8 (0.7–1.0)	<0.01	0.2 (-0.3–0.8)	0.4	1.3 (1.0–1.5)	<0.05
Dyslipidemia	1.4 (1.1–1.8)	0.01	1.5 (1.2–1.9)	0.001	1.2 (1.0–1.4)	0.11	1.1 (1.0–1.4)	0.2	01.0 (0.2–1.8)	<0.05	1.1 (0.8–1.4)	0.7
Hyperlipidemia	0.9 (0.7–1.1)	0.25	0.8 (0.7–1.0)	0.03	0.9 (0.8–1.0)	0.05	0.9 (0.8–1.0)	<0.05	-0.7 (-1.2–0.1)	<0.05	1.1 (0.9–1.3)	0.4
Hypertension	1.1 (0.9–1.4)	0.45	1.4 (1.1–1.8)	<0.01	1.2 (1.0–1.5)	0.05	1.2 (1.0–1.4)	0.1	0.7 (-0.1–1.4)	0.1	1.3 (1.0–1.7)	<0.05
Hypothyroidism	1.0 (0.8–1.2)	0.75	1.0 (0.8–1.3)	0.82	1.1 (0.9–1.3)	0.40	0.9 (0.8–1.0)	<0.05	0.3 (-0.3–0.9)	0.4	1.0 (0.8–1.2)	1.0
Ischemic heart	1.1 (0.8–1.5)	0.52	1.1 (0.9–1.5)	0.4	1.3 (1.0–1.6)	0.04	0.9 (0.7–1.1)	0.4	0.1 (-0.9–1.1)	0.9	1.2 (0.9–1.7)	0.3
Insomnia	0.7 (0.5–0.9)	0.01	0.8 (0.6–1.1)	0.2	0.9 (0.7–1.2)	0.42	1.0 (0.8–1.2)	0.6	0.9 (0.1–1.7)	<0.05	1.1 (0.8–1.5)	0.4
Defibrillator	0.6 (0.5–0.9)	0.01	0.4 (0.3–0.6)	<0.0001	0.6 (0.4–0.8)	<0.001	0.6 (0.5–0.8)	<0.001	-2.2 (-3.4–1.1)	<0.001	1.0 (0.7–1.5)	1.0
Not resuscitate	28.1 (23.2–34.0)	<0.0001	7.3 (6.1–8.7)	<0.0001	4.1 (3.6–4.8)	<0.0001	2.8 (2.5–3.2)	<0.0001	2.9 (2.3–3.6)	<0.0001	1.0 (0.8–1.2)	0.9
Acute myocardial infarction	1.3 (1.0–1.7)	0.10	1.3 (1.0–1.8)	<0.05	1.3 (1.0–1.6)	0.03	1.5 (1.3–1.98)	<0.0001	1.7 (0.7–2.6)	<0.001	0.9 (0.7–1.2)	0.5
Atrial fibrillation	1.5 (1.3–1.9)	<0.0001	1.3 (1.1–1.6)	<0.05	1.2 (1.0–1.5)	0.03	1.0 (0.8–1.2)	0.8288	0.7 (-0.1–1.58)	0.1	0.9 (0.7–1.2)	0.4
Ventricular fibrillation	1.8 (1.3–2.5)	<0.001	1.7 (1.2–2.3)	0.001	1.7 (1.3–2.2)	<0.0001	1.5 (1.2–1.9)	0.001	1.9 (0.7–3.1)	<0.01	0.9 (0.6–1.2)	0.4
Morbid obesity	1.5 (1.2–1.8)	<0.0001	1.8 (1.5–2.1)	<0.0001	1.5 (1.3–1.79)	<0.0001	1.5 (1.3–1.6)	<0.0001	1.5 (1.0–2.0)	<0.0001	1.0 (0.8–1.1)	0.6
Sleep apnea	1.2 (0.9–1.5)	0.22	1.2 (1.0–1.5)	0.1	1.2 (1.0–1.4)	0.1	1.0 (0.8–1.2)	0.9	0.8 (0.1–1.6)	<0.05	1.0 (0.8–1.4)	0.8
Obesity hypoventilation	0.8 (0.4–1.7)	0.6	1.3 (0.8–2.3)	0.4	1.3 (0.8–2.0)	0.4	0.7 (0.4–1.0)	0.06	1.8 (-0.2–3.7)	0.1	1.3 (0.6–2.6)	0.5
**Covid-19 symptoms**												
Cough	1.0 (0.8–1.2)	1.0	1.0 (0.8–1.1)	0.6	0.9 (0.8–1.0)	0.2	0.9 (0.8–1.1)	0.3	-0.6 (-1.1–0.1)	<0.05	4.9 (3.7–6.4)	<0.0001
Abdominal symptoms	0.7 (0.5–1.0)	<0.05	0.78 (0.6–1.0)	<0.05	0.8 (0.7–1.0)	<0.05	0.8 (0.7–1.0)	<0.05	-0.9 (-1.5–0.2)	<0.01	12.5 (7.7–20.4)	<0.0001
Dyspnea	1.3 (1.1–1.5)	<0.01	1.2 (1.0–1.5)	<0.05	1.2 (1.0–1.3)	<0.05	1.1 (1.0–1.3)	<0.05	0.3 (-0.2–0.7)	0.2	8.8 (7.2–10.8)	<0.0001
Fever	1.1 (0.96–1.3)	0.6	1.0 (0.8–1.2)	1.0	1.0 (0.9–1.2)	0.9	1.4 (1.2–1.6)	<0.0001	0.3 (-0.2–0.8)	0.2	8.7 (6.3–12.0)	<0.0001
Admitted through ED	0.9 (0.7–1.2)	0.6	0.6 (0.5–0.8)	<0.0001	0.7 (0.6–0.8)	<0.0001	1.2 (1.0–1.3)	0.1	-1.6 (-2.2–1.0)	<0.0001	NA	NA

Cont., continuous variable; OR, odds ratio; Coef, coefficient; RF, reference; CI, confidence interval; NA, not available.

### Factors associated with COVID-19 outcomes using multivariable analyses

#### Sepsis, ICU admission, mechanical ventilation, and hospital mortality

Multivariable analyses indicated that after controlling for other factors, including a proportion of inter-hospital transfers, Native American patients had a greater chance of dying in the hospital (OR, 2.3; 95%CI, 1.6–3.2), receiving invasive mechanical ventilation (OR, 2.3; 95%CI, 1.8–3.0), being admitted to the ICU (OR, 2.4; 95%CI, 1.9–3.0), and developing sepsis (OR, 1.7; 95%CI, 1.4–2.8) (all *P* < 0.05; Table 3). Hispanic patients had a greater risk of hospital mortality (OR, 1.4; 95%CI, 1.1–1.7), mechanical ventilation (OR, 1.4; 95%CI, 1.2–1.6), and sepsis (OR, 1.2; 95%CI, 1.1–1.3) than non-Hispanic white individuals (all *P* < 0.05; Table 3). Male patients had an increased risk for hospital death (OR, 1.7; 95%CI, 1.4–2.0), invasive mechanical ventilation (OR, 2.0; 95%CI, 1.7–2.3), ICU admission (OR, 1.7; 95%CI, 1.5–1.9), and sepsis (OR, 1.4; 95%CI, 1.3–1.6) (all *P* < 0.0001; Table 3). Older age only significantly increased the odds of hospital death (OR, 1.02; 95%CI, 1.01–1.04; *P* < 0.0001; Table 3). Having Medicaid or Medicare compared to having private insurance was not significantly associated with any outcomes. However, residence in high-income areas was associated with lower odds of hospital death (OR, 0.4; 95%CI, 0.2–0.9), ventilation (OR, 0.2; 95%CI, 0.1–0.5), and ICU admission (OR, 0.3; 95%CI, 0.27–0.6) (all *P* < 0.05; Table 3). Chronic conditions such as lung diseases, diabetes, ventricular fibrillation, obesity, and symptoms such as dyspnea were associated with increased odds of hospital death, ventilation, ICU admission, and sepsis (all OR > 1 and *P* < 0.05). Patients admitted through the ED had lower odds of ventilation (OR, 0.63; 95%CI, 0.52–0.77; *P* < 0.0001) and ICU admission (OR, 0.69; 95%CI, 0.59–0.81; *P* < 0.0001).

#### Hospital stay and admission through ED

Native American race and Hispanic ethnicity were associated with longer LOS by 2.06 days (coefficient, 2.06; 95%CI, 1.16–2.96; *P* < 0.0001) and 0.48 days (coefficient, 0.48, 95%CI, 0.06–0.96; *P* < 0.0001) compared with non-Hispanic white individuals. Medicaid insurance was not associated with LOS when compared to commercial plans while increasing income was associated with shorter LOS (coefficient, −3.0; 95%CI, −5.2–0.8), *P* < 0.01; Table 3; *P* < 0.01). Male sex (coefficient,1.2; 95%CI, 0.8–1.6, *P* < 0.0001), older age (coefficient,0.1; 95%CI, 0.1–0.1; *P* < 0.0001), chronic lung conditions (coefficient, 11.9; 95%CI, 9.7–14.0), and kidney conditions (coefficient; 1.5; 95%CI, 0.9–2.1) increased LOS (all *P* < 0.0001; Table 3). However, admission through the ED was associated with shorter LOS (coefficient, −1.6; 95%CI, −2.2—1.0; *P* < 0.0001; Table 3).

Factors associated with ED admission were also investigated. Native American patients had lower odds of admission through the ED than white individuals (OR, 0.17; 95%CI, 0.13–0.22; *P* < 0.0001), while Black individuals had higher odds (OR,1.69; 95%CI, 1.21–2.36, *P* < 0.01) than non-Hispanic white individuals (Table 3).

#### ICU stay and time to ICU

Within the ICU subgroup, chronic lung diseases (coefficient, 4.4; 95%CI, 0.0–8.8) and presenting symptoms such as fever (coefficient,1.7; 95%CI, 0.1–3.3) were associated with a longer ICU LOS (all *P* < 0.05; Table 4). Having Medicare (coefficient, −2.3; 95%CI, −4.2–0.4; *P* < 0.05) and being admitted through the ED (coefficient, −2.6; 95%CI, −3.9–1.47; *P* < 0.0001) were associated with a shorter ICU LOS (Table 4). Native American race was highly associated with a shorter time from registration to ICU admission after controlling for all covariates including being admitted to the ICU through the ED (coefficient, −20.4; 95%CI, −35.4–5.3; *P* < 0.01; Table 4).

**Table 4:** Multivariable linear regression results for factors associated with ICU length of stay and time from registration to ICU admission.

	**Total length of ICU stay**		**Hours from registration to ICU**	
	**Coef (95%CI)**	***P-*** **values**	**Coef (95%CI)**	***P-*** **values**
Age (Cont.)	0.0 (−0.1–0.1)	1.0	0.4 (0.1–0.8)	0.02
**Race**				
White	RF	NA	RF	NA
Native American	1.7 (−0.5–3.9)	0.1	−20.4 (−35.4–5.3)	<0.01
Black or African American	−1.0 (−4.0–1.9)	0.5	10.6 (−11.0–32.2)	0.3
Hispanic	1.3 (−0.2–2.8)	0.1	5.7 (−4.6–16.0)	0.3
Others	1.0 (−1.7–3.7)	0.5	−20.6 (−39.3–2.0)	<0.05
**Insurance**				
Private	RF	NA	RF	NA
Medicaid	0.4 (−1.3–2.1)	0.6	−4.9 (−17.6–7.8)	0.5
Medicare	−2.3 (−4.2–0.4)	<0.05	−2.9 (−16.2–10.3)	0.7
Others	−0.6 (−10.8–9.6)	0.9	−16.8 (−108.6–75.0)	0.7
Self–Pay	−3.1 (−5.7–0.6)	<0.05	−15.7 (−35.4–4.1)	0.1
Male vs. female	0.6 (−0.7–1.9)	0.4	5.2 (−3.7–14.1)	0.3
Standardized income (Cont.)	−0.5 (−8.0–7.0)	0.9	48.8 (−0.2–97.7)	0.05
**Chronic conditions**				
Stroke	1.9 (−0.5–4.3)	0.1	−5.3 (−19.0–8.4)	0.5
Chronic kidney	1.6 (−0.1–3.3)	0.1	16.1 (5.3–26.9)	<0.01
Chronic liver	−1.9 (−4.6–0.8)	0.2	4.5 (−12.8–21.8)	0.6
Hypoxemia	−4.5 (−5.8–3.2)	<0.0001	−1.0 (−9.7–7.8)	0.8
Chronic lung	4.4 (0.0–8.8)	0.04	45.7 (26.7–64.8)	<0.0001
Coronary heart	−1.7 (−3.8–0.3)	0.1	4.9 (−7.7–17.5)	0.4
Diabetes mellitus	1.7 (0.4–3.0)	<0.05	2.3 (−6.7–11.3)	0.6
Dementia	−4.6 (−7.6–1.6)	<0.01	−5.0 (−22.1–12.0)	0.6
Depression	−0.2 (−1.8–1.4)	0.8	5.8 (−5.4–16.9)	0.3
Hyperlipidemia	−1.5 (−3.0–0.0)	0.05	4.2 (−5.9–14.4)	0.4
Hypertension	1.6 (−0.4–3.6)	0.1	0.91 (−11.6–13.5)	0.9
Hyperthyroidism	−3.6 (−9.0–1.8)	0.2	−1.7 (−42.2–38.8)	0.9
Hypothyroidism	−0.3 (−2.2–1.5)	0.7	−4.2 (−16.0–7.7)	0.5
Dyslipidemia	3.7 (1.5–6.0)	<0.001	−7.5 (−21.6–6.7)	0.3
Insomnia	1.1 (−1.3–3.6)	0.4	19.8 (2.2–37.5)	<0.05
Ischemic heart	0.2 (−2.5–2.9)	0.9	−3.7 (−19.8–12.4)	0.7
Defibrillator	−4.5 (−8.2–0.9)	<0.05	1.8 (−20.5–24.1)	0.9
Not resuscitate	4.5 (2.7–6.4)	<0.0001	10.1 (0.5–19.6)	<0.05
Acute myocardial infarction	1.4 (−1.0–3.9)	0.2	−6.6 (−21.7–8.6)	0.4
Atrial fibrillation	2.7 (0.7–4.9)	<0.05	6.3 (−5.7–18.3)	0.3
Ventricular fibrillation	−0.5 (−3.5–2.5)	0.7	10.2 (−6.6–27.0)	0.2
Morbid obesity	2.0 (0.6–3.4)	<0.01	−0.5 (−10.2–9.2)	0.9
Sleep apnea	0.1 (−1.8–2.0)	1.0	−10.4 (−23.4–2.6)	0.1
**Symptoms**				
Cough	−1.5 (−3.1–0.1)	0.1	10.3 (−0.7–21.3)	0.1
Abdominal symptoms	−0.8 (−2.9–1.4)	0.5	6.7 (−8.9–22.2)	0.4
Dyspnea	0.6 (−0.8–1.9)	0.4	11.4 (2.2–20.6)	<0.05
Fever	1.7 (0.1–3.3)	0.03	10.0 (−0.8–20.9)	0.1
Admitted from ED to ICU	−2.6 (−3.9–1.47)	<0.0001	−81.1 (−89.9–72.3)	<0.0001

Cont., continuous variable; Coef, coefficient; RF, reference; CI, confidence interval; NA, not available.

## Discussion

This retrospective cohort study characterized the COVID-19 health disparities and patient outcomes in Native American individuals within a large healthcare system in the Southwest US. Although many small-scale studies have investigated similar health disparities, to our knowledge, this is the first comprehensive study with sufficient representation of the Native American population that was performed to measure case-fatality rates. Our results showed that Native American, Black individuals, and Hispanic patients were disproportionately burdened with a greater proportion of hospitalization when compared to race/ethnicity distribution based upon census data (24). These findings are consistent with findings in other studies and may be attributable to high infection rates in these minorities secondary to social determinants of health and other reasons (25, 26). However, in such studies mortality was based upon data from the National Death Index of the CDC, and case-fatality rates and hospital course of these individuals were not assessed (12). Conceivably, a greater number of infections in the minority populations could have explained the greater mortality in Native American individuals. Our findings align with a recent report that used Agency for Healthcare Research and Quality (AHRQ) 2020 NIS dataset for hospitalizations from 1 January 2020 to 31 December 2020 to show that Native American men had the highest inpatient mortality due to COVID-19 (11). To our knowledge, our study is the first to demonstrate a greater case-fatality rate in a hospitalized setting among Native Americans irrespective of gender after adjusting for multiple covariates.

In our study, the greater prevalence of chronic lung disease (OR 6.14; 95%CI; 3.91–9.63; Table 3) may be responsible for the observed greater hospital mortality in Native American individuals when compared to non-Hispanic white individuals or even other minorities. Previously, investigators have found that patients with chronic lung disease (e.g., chronic obstructive pulmonary disease) had the highest odds of COVID-19 mortality compared with patients with other pre-existing cardiometabolic conditions, such as obesity, diabetes, and hypertension (27). Moreover, the Native American populations have a higher adjusted prevalence of chronic respiratory disease compared with the non-Hispanic white population (28). Such differences in the presence of chronic lung disease as comorbidity are important information that healthcare providers and systems need to consider when caring for Native American individuals or even other patients.

Information on racial disparities in COVID-19 outcomes has largely come from studies on the Black population, with minimal research on the impact on Native American individuals (12). Some studies reveal that Native American individuals experienced disproportionate infection and mortality rates during the COVID-19 pandemic (10). Our study furthers our understanding of such findings by demonstrating the greater likelihood of hospital death, invasive ventilation, ICU admission, and sepsis in Native American individuals, even after adjusting for differences in sociodemographic factors (insurance status and median household income) and other clinical characteristics. Interestingly, while older age and male sex are commonly reported risk factors for adverse COVID-19 outcomes, the median age of Native American patients in our study was 17 years younger than in non-Hispanic white individuals with a near 1:1 ratio between men and women (29). Thus, age and sex did not contribute to the observed adverse COVID-19 outcomes in Native American individuals.

Observed associations between Native American race and COVID-19 outcomes may be multifactorial. One factor may be racial differences in immune response (30). Studies have found that Native American individuals have genetic susceptibility and subdued innate response to novel viral infection (31). Another factor may be related to the lower rates of common COVID-19 symptoms in Native American individuals than non-Hispanic white individuals. The racial difference in symptom presentation may reflect a difference in the underlying immune response or end-organ response to COVID-19 and may lead to delays in seeking medical attention. Such atypical clinical presentation and symptomatology of COVID-19 in Native American patients may increase the difficulty for patients and healthcare providers to identify COVID-19 and monitor clinical changes. Such factors may also underlie greater case-fatality rates in Native American individuals, highlighting the necessity of increased precautions when caring for Native American patients with COVID-19. As COVID-19 is still a novel viral respiratory infection, its epidemiology and clinical responses are yet unknown among different racial groups, and more studies are needed to assess racial differences in immune response and clinical presentation.

Factors such as social determinants of health may also contribute to the observed racial differences in COVID-19 outcomes. One in four Native American individuals is in poverty with a median annual income below the national average and is more likely to have chronic diseases (7, 32). The median income of residence areas for Native American patients was significantly lower than white individuals, and a greater share of Native American individuals had Medicaid than any other racial group, with 79% of Native American individuals between ages 18 and 39 on Medicaid. Consistent with national surveys, Native American inpatients had higher rates of diabetes, obesity, and chronic lung disease than non-Hispanic white individuals (7). Studies in other races have reported associations between social inequalities in housing, education level, and jobs, and the susceptibility to COVID-19 (33). Many long-standing socioeconomic issues such as living conditions, transportation, employment, access to health services, and running water affect Native American individuals as well (34). These health and socioeconomic determinants may have made Native American individuals more vulnerable to COVID-19. However, no strong association was found between the Native American race and the number of comorbidities in our study, although individual conditions such as chronic lung and liver disease were associated with greater risk for mortality. Such findings further underscore the importance of understanding organ-specific mechanisms—such as lung and liver—that may be responsible for the observed difference. Interestingly, certain single nucleotide polymorphisms (SNPs; rs2070788 and rs383510) that were associated with severe pulmonary damage caused by influenza A(H7N9) in 2014 and influenza A(H1N1) in 2009 are surprisingly the highest in Native American individuals in a worldwide study (35).

In our study, only 43% of Native American individuals were admitted through the emergency department (ED), whereas more than 80% of Black, Hispanic and non-Hispanic white individuals were admitted through ED. The low rate of hospitalization through the ED was carried across all age cohorts of Native American individuals and remained independent of sociodemographic factors and clinical presentation. Previous studies have found that older individuals, men, Black individuals, and low-income populations had higher rates of visiting the ED (36). In keeping with such findings, our study showed that the older adults, men, Black individuals, individuals from lower socioeconomic status, and individuals with chronic liver disease, hypertension, dementia, depression, and symptoms were more likely to be admitted through the ED. But the finding that a majority of Native American individuals were inter-hospital transfers may be reflective of barriers to accessing higher level care and adjusted for in our analyses of other outcomes such as mortality and time to ICU admission. However, it is well known that inter-hospital transfer is independently associated with greater in-hospital mortality, especially from rural areas (37). While such findings suggest issues with access to care, delayed presentation, and destabilization during limited treatment availabilities during transit, we observed that the greater in-hospital mortality in Native American individuals when compared to non-Hispanic white individuals remained even after adjusting for inter-hospital transfers (Table 3). Such findings suggest that other factors may be responsible for the observed greater mortality in Native American individuals.

We investigated the relationship between time from hospital admission to ICU transfer as a surrogate measure of the rate of COVID-19 progression and clinical deterioration. Our multivariable analysis revealed that the time to ICU transfer in Native American individuals was shorter than in other racial groups across all age groups. These findings suggest that Native American individuals with COVID-19 progressed more rapidly than others. We recognize that we do not have time spent by patients at outside hospitals when they underwent inter-hospital transfers, and that is a limitation. However, despite adjusting for inter-hospital transfer status, we found that the time to deterioration was greater in Native American individuals. Moreover, an increased odd of admission from the ED to the ICU was noted in Native American individuals (Table 4), which was not affected by inter-hospital transfer status or time spent on the floor in the outside hospital. To our knowledge, our study is the first to investigate the clinical deterioration rates in COVID-19 inpatients and higher case-fatality rates for various race and ethnicity groups. In combination with the lower rate of commonly reported symptoms of COVID-19 in Native American individuals, these results may emphasize the importance of closer monitoring of Native American patients upon entry or admission to the hospital.

Our study has several limitations, the first being the retrospective observational nature of the data. Therefore, adjustment for all confounders was not possible. Our study sample has a better representation of Native American populations because Banner Health hospitals serve as the tertiary care referral centers for many critical access hospitals in the southwestern United States. Therefore, it is possible that we missed a large proportion of the Native American population, hence limiting the generalizability of our findings. However, our large sample size and detailed phenotyping allowed adjustment for multiple covariates.

## Public health implications

The greater case-fatality rates following hospitalization in Native American individuals emphasize the systematic disadvantage experienced by Native American individuals and point out the need to address health inequalities by developing programs that improve overall health outcomes. Atypical symptom presentation of COVID-19, greater prevalence of chronic lung disease, and more rapid clinical deterioration than other races/ethnicities underscore the role of pulmonologists in addressing such disparities. The association between greater COVID-19-related mortality and chronic lung disease is being addressed by improving COVID-19 vaccination rates in adults in the pulmonary ambulatory setting (38–40).

## Data availability statement

The original contributions presented in the study are included in the article/Supplementary material, further inquiries can be directed to the corresponding author.

## Ethics statement

The studies involving human participants were reviewed and approved by Banner Health IRB 483-20-0076. Written informed consent for participation was not required for this study in accordance with the national legislation and the institutional requirements.

## Author contributions

CB, YW, GC, DS, SK, PT, and SP participated in the conception of the work, planning, data collection, analysis and manuscript writing and review of final draft. VK participated in data analysis, manuscript writing and review of final draft. All authors contributed to the article and approved the submitted version.
