# Supplementary material for: Disparities in outcomes of COVID-19 hospitalizations in native American individuals
Source: Front Public Health. 2023 Aug 15;11:1220582. doi: 10.3389/fpubh.2023.1220582 (PMC10465166; doi:10.3389/fpubh.2023.1220582)
Supplement: Supplementary file 3 [file Data_Sheet_1.PDF]

SUMPLIMENTARY FIGURES

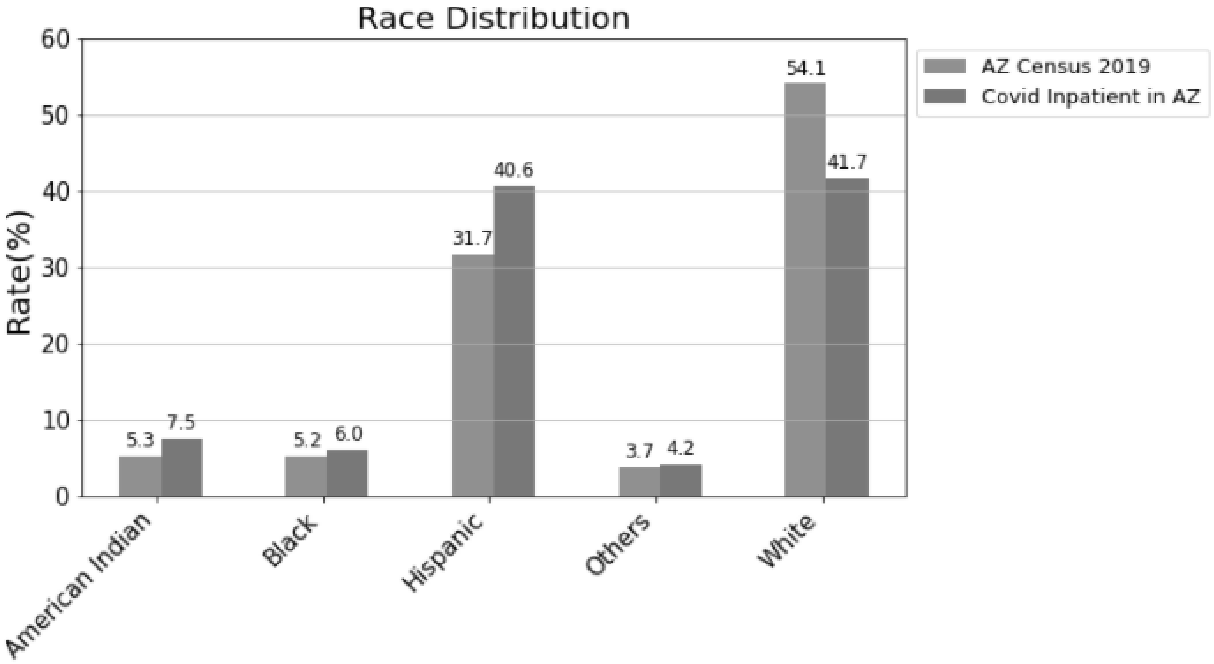

Fig. S1 Distribution of race in Covid inpatients in AZ compared to AZ census data 2019

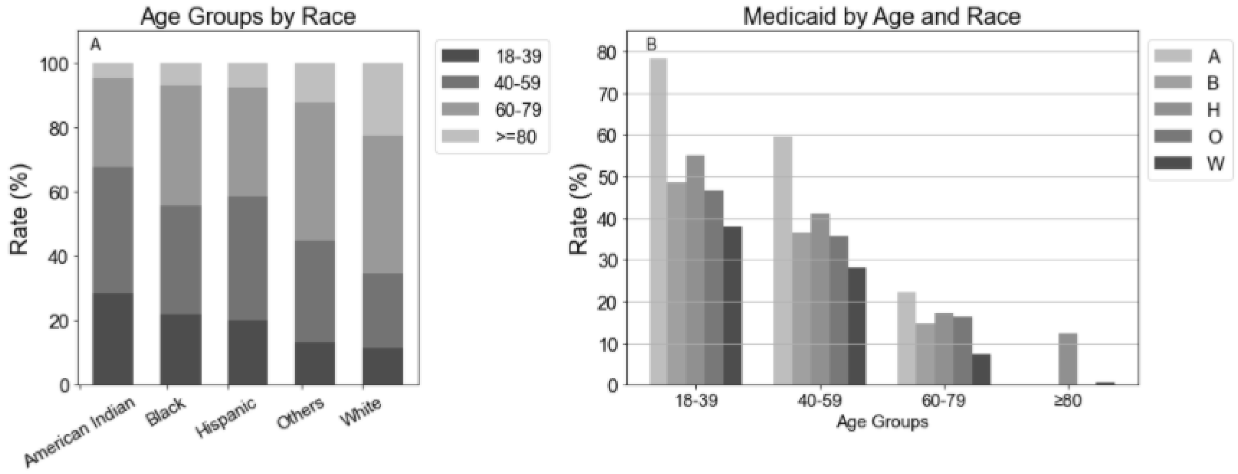

Fig. S2 A Distribution of Age Groups by Race in Hospitalized Population; B: Distribution of Medicaid by Age and Race in Hospitalized Population; Note: ‘A’, Native American; ‘B’, Black and African American; ‘H’, Hispanic; ‘O’, Others; ‘W’, White/Caucasian

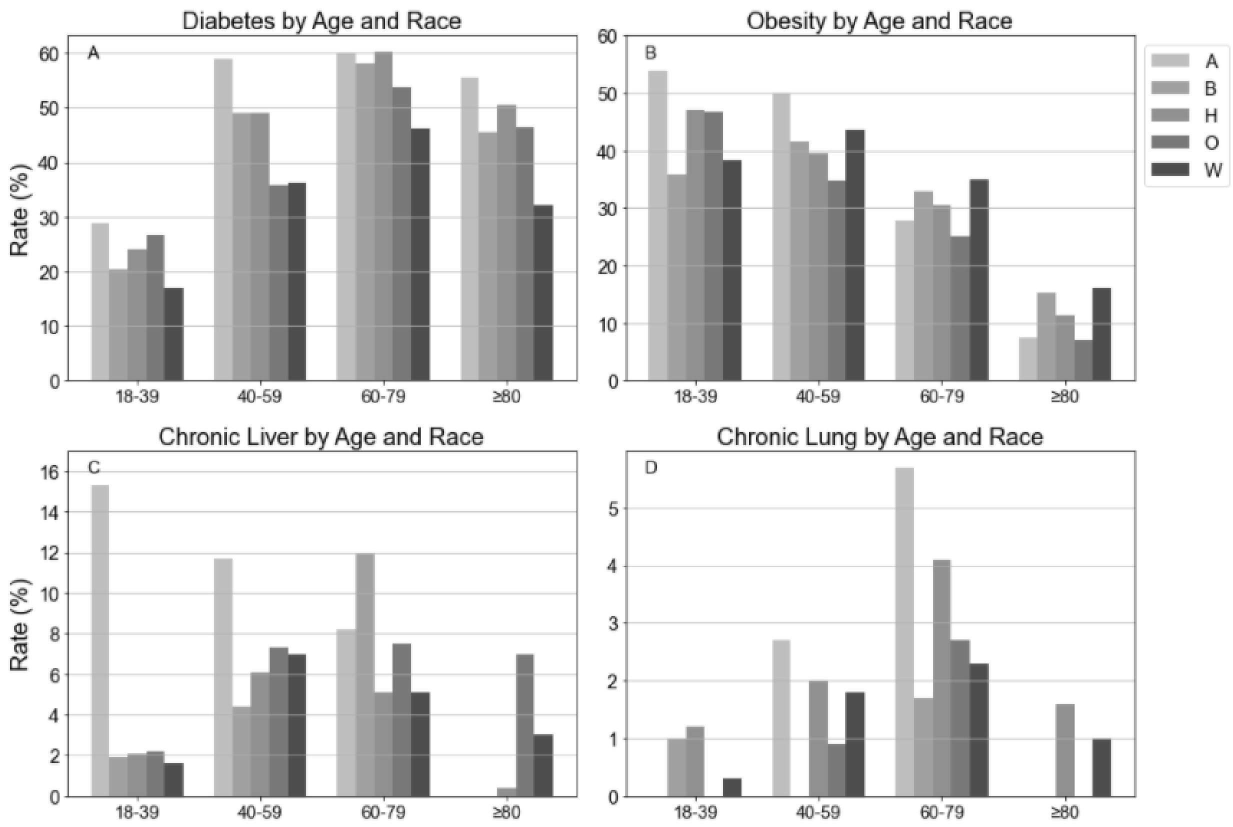

Fig. S3 Distributions of Chronic Condisitons by Age and Race in Hospitalized Population. Note: ‘A’, Native American; ‘B’, Black and African American; ‘H’, Hispanic; ‘O’, Others; ‘W’, White/Caucasian
